# Transplantation of human induced pluripotent stem cell-derived neural crest cells for corneal endothelial regeneration

**DOI:** 10.1186/s13287-021-02267-z

**Published:** 2021-03-29

**Authors:** Yajie Gong, Haoyun Duan, Xin Wang, Can Zhao, Wenjing Li, Chunxiao Dong, Zongyi Li, Qingjun Zhou

**Affiliations:** 1 Shandong First Medical University & Shandong Academy of Medical Sciences, 6699 Qingdao Road, Jinan, 271016 China; 2 State Key Laboratory Cultivation Base, Shandong Provincial Key Laboratory of Ophthalmology, Shandong Eye Institute, Shandong First Medical University & Shandong Academy of Medical Sciences, 5 Yan’erdao Road, Qingdao, 266071 China; 3 Eye Hospital of Shandong First Medical University, 372 Jingsi Road, Jinan, 250021 China

**Keywords:** iPSC, Neural crest cells, Corneal endothelium, Transplantation

## Abstract

**Background:**

The corneal endothelium maintains corneal hydration through the barrier and pump function, while its dysfunction may cause corneal edema and vision reduction. Considering its development from neural crest cells (NCCs), here we investigated the efficacy of the human induced pluripotent stem cell (hiPSC)-derived NCCs for corneal endothelial regeneration in rabbits.

**Methods:**

Directed differentiation of hiPSC-derived NCCs was achieved using the chemically defined medium containing GSK-3 inhibitor and TGF-β inhibitor. The differentiated cells were characterized by immunofluorescence staining, FACS analysis, and in vitro multi-lineage differentiation capacity. For in vivo functional evaluation, 1.0 × 10^6^ hiPSC-derived NCCs or NIH-3 T3 fibroblasts (as control) combined with 100 μM Y-27632 were intracamerally injected into the anterior chamber of rabbits following removal of corneal endothelium. Rabbit corneal thickness and phenotype changes of the transplanted cells were examined at 7 and 14 days with handy pachymeter, dual-immunofluorescence staining, and quantitative RT-PCR.

**Results:**

The hiPSC-derived NCCs were differentiated homogenously through 7 days of induction and exhibited multi-lineage differentiation capacity into peripheral neurons, mesenchymal stem cells, and corneal keratocytes. After 7 days of intracameral injection in rabbit, the hiPSC-derived NCCs led to a gradual recovery of normal corneal thickness and clarity, when comparing to control rabbit with fibroblasts injection. However, the recovery efficacy after 14 days deteriorated and caused the reappearance of corneal edema. Mechanistically, the transplanted cells exhibited the impaired maturation, cellular senescence, and endothelial-mesenchymal transition (EnMT) after the early stage of the in vivo directional differentiation.

**Conclusions:**

Transplantation of the hiPSC-derived NCCs rapidly restored rabbit corneal thickness and clarity. However, the long-term recovery efficacy was impaired by the improper maturation, senescence, and EnMT of the transplanted cells.

## Introduction

The corneal endothelium is a monolayer of hexagonal cells located on the posterior surface that separates corneal stroma from the aqueous humor of anterior chamber. It maintains normal corneal hydration and clarity through the pump and barrier function [1, 2]. The regenerative capacity of human corneal endothelial cells (CECs) is extremely restricted in vivo. Once damaged, the loss area can only be compensated by the enlargement of remnant endothelial cells [3]. Therefore, the sufficient cell density is critical for the functional maintenance of corneal endothelium. When the density decreases lower than the critical level (< 400–500 mm^2^) due to eye surgery, dystrophy, trauma, toxicity, inflammation, high intraocular pressure, and various pathological stimuli, the corneal endothelium will develop decompensation and cannot maintain the stromal hydration, resulting in corneal edema and even vision loss, known as bullous keratopathy in clinic [4–6].

Corneal transplantation, such as penetrating keratoplasty, Descemet’s stripping automated endothelial keratoplasty, and Descemet membrane endothelial keratoplasty, is currently dominant therapeutic treatment for the irreversible corneal endothelial decompensation [7]. However, all approaches rely on the sufficient support of donor corneas with high quality of corneal endothelium, which were severely limited worldwide. Furthermore, the various complications and allogeneic graft rejection remained as the major issues of corneal transplantation [7]. Recently, the intracameral injection of cultured CECs with the ROCK inhibitor Y-27632 was successfully developed and followed 5 years for the treatment of bullous keratopathy [5, 8]. In addition, multiple alternative stem cells, such as mesenchymal stem cells [9], skin-derived precursors [10], adipose-derived stem cells [11], and corneal stromal stem cells [12] may represent the suitable for CEC therapies. However, the immune rejection, ethical problems, and limited tissue accessibility still hamper the clinical application.

Human induced pluripotent stem cells (hiPSCs) represent a more potential source of various terminally differentiated cells in regenerative medicine when compared to human embryonic stem cells (hESCs), because of the autogenous transplantation potential [13]. Through the recapitulation of developmental process, the two-stage inductive methods had been reported to induce hiPSCs or hESCs into corneal endothelial-like cells via the middle stage of neural crest cells (NCCs) [14, 15]. However, the differentiation strategies of the corneal endothelial-like cells from NCCs mostly rely on co-culture and the use of conditioned media, since the specific marker of CECs and regulatory pathway of inducing proper differentiation remains unclear or signal pathway of CEC differentiation remains unclear [14, 16]. Comparatively, the homogenous induction of NCCs from hiPSCs had been described with about 90% differentiation efficiency by inhibiting TGF-β-Smad signaling and activating Wnt signaling [17]. The neural crest-derived cells possessed the capacity of regenerating bone and dental tissue [18, 19], as well as for the treatment of Parkinson’s disease and spinal cord injury [20, 21]. Therefore, human induced pluripotent stem cell-derived neural crest cells (hiPSC-derived NCCs) may serve as a novel cell source for corneal endothelial regeneration.

In this study, we induced the direct differentiation of NCCs from hiPSCs according to the classical method of using GSK-3 and TGF-β inhibitors. When injected into the anterior chamber of rabbit model, the hiPSC-derived NCCs differentiated into the corneal endothelial-like cells and recovered the normal corneal clarity and thickness within the first 7 days. However, the transplanted cells gradually underwent improper maturation, cellular senescence, and endothelial-mesenchymal transition (EnMT), which finally led to the reappearance of corneal edema and clarity loss.

## Materials and methods

### Animals

New Zealand white rabbits (XiLingjiao, Jinan, China) were housed and treated following the Association for Research in Vision and Ophthalmology (ARVO) Statement for the Use of Animals in Ophthalmic and Vision Research. All animal experiments were approved by the Ethics Committee of Shandong Eye Institute.

### Cell culture

The hiPSC U2 line (Cellapybio, Beijing, China) was cultured on Matrigel-coated (Corning, Corning, NY, USA) dishes and maintained in stem cell medium (SCM) called mTeSR1 medium (STEMCELL Technologies, Vancouver, BC, Canada) in a 37 °C incubator with 5% CO_2_. NIH-3 T3 fibroblasts (Shanghai Cell Bank of Chinese Academy of Sciences, Shanghai, China) was cultured with Dulbecco’s modified Eagle medium (DMEM; Corning) containing 10% fetal bovine serum (FBS; Gibco, Grand Island, NY, USA) and 1% penicillin/streptomycin (PenStrep; Corning). The medium was changed every 2 days and cells were passaged twice a week [22].

### Neural crest cell and peripheral neuron differentiation

The NCCs were differentiated from hiPSCs according to the previously described method [17, 23]. Briefly, the hiPSCs were transferred to a neural crest differentiation medium (NDM) containing DMEM/F12 medium (Gibco), 2% bovine serum albumin (BSA; Sigma, St. Louis, MO, USA), 200 ng/mL human insulin-like growth factor 1 (PeproTech, NJ, USA), 10 ng/mL Heregulinβ-1 (PeproTech), 8 ng/mL fibroblast growth factor 2 (FGF2; R&D Systems, Minneapolis, MN, USA), 50 μg/mL sodium L-ascorbic acid salt (Sigma), 1% insulin-transferrin-selenium solution (ITS; Gibco), 1% MEM non-essential amino acids solution (MEM-NEAA; Gibco), 0.1 mM 2-mercaptoethanol (Sigma), 2 mM L-GlutaMAX (Gibco), 2.0 μM SB431542 (EMD Millipore Corporation, Burlington, MA, USA), 1.0 μM CHIR99021 (Med Chem Express, NJ, USA), 1.0 μM DMH1 (Sigma), and 15 ng/mL bone morphogenetic protein 4 (BMP4; PeproTech) for 7 days. The medium was changed every day.

Peripheral neurons were differentiated by plating the hiPSC-derived NCCs in poly-L-ornithine/Laminin-coated (Sigma) dishes and culturing in peripheral neuron differentiation medium containing DMEM/F12, N2 supplement (Gibco), 10 ng/mL brain-derived neurotrophic factor (R&D Systems), 10 ng/mL glial cell line-derived growth factor (R&D Systems), 10 ng/mL nerve growth factor (PeproTech), 10 ng/mL neurotrophin-3 (PeproTech), 200 μM sodium L-ascorbic acid salt, and 0.5 mM dbcAMP (Sigma). The cells were incubated for 12–14 days with the medium changed every 2 days.

### Mesenchymal cell and corneal keratocyte differentiation

For mesenchymal differentiation, the hiPSC-derived NCCs were cultured in medium containing DMEM/F12, 10% FBS, 1% PenStrep, 1% l-alanyl-l-glutamine (Gibco), and 0.1 mM 2-mercaptoethanol and passaged every 4–5 days [17]. Adipogenic differentiation was induced by treating the induced mesenchymal cells with the MesenCult™ Adipogenic Differentiation Kit (STEMCELL Technologies) according to the manufacturer’s protocol, with medium replacement every 3 days. After 3 weeks, the cells were fixed with 10% neutral buffered formalin (Sigma) for 10 min at room temperature and stained with oil red O (Sigma). The osteogenic differentiation capacity of the hiPSC-NCCs was confirmed using the MesenCult™ Osteogenic Differentiation Kit (STEMCELL Technologies). The medium was changed every 3 days and the cells were cultured for 10–15 days, followed by fixation with 70% ethanol solution for 1 h and stained with 0.1% Alizarin Red S (Sigma). Chondrogenic differentiation was elicited by treating the induced mesenchymal cells with MesenCult™-ACF Chondrogenic Differentiation Kit (STEMCELL Technologies) for 3–4 weeks, with medium changes every 2 days. The cells were then fixed with 10% neutral buffered formalin and stained with 1% Alcian Blue (Sigma) [24]. For corneal keratocyte differentiation, the hiPSC-derived NCCs were plated in Matrigel-coated plates, followed by exposure to keratocyte differentiation medium consisting of DMEM/F12, 10 ng/mL FGF2, 1 mM ascorbic acid-2-phosphate (Sigma), 1% ITS, and 1% MEM-NEAA [25].

### Establishment of rabbit corneal endothelial dysfunction model

A total of 42 rabbits were randomly divided into two groups: the hiPSC-derived NCCs group (*n* = 39) and the NIH-3 T3 fibroblasts group as a control (*n* = 3). The rabbits were anesthetized with pentobarbital sodium (50 mg/kg, Shanghai, China) by intravenous injection. A rabbit corneal endothelial dysfunction model was performed according to a previously described method [26, 27]. Briefly, the right eye of each rabbit was damaged to mimic corneal endothelial dysfunction. Then, the anterior chamber was punctured with a corneal knife at the corneal limbus. Sodium hyaluronate was injected through the puncture with a 27-G blunt needle to maintain a space in the anterior chamber. The corneal endothelium was mechanically scraped from Descemet’s membrane with a 20-G soft tapered silicone needle (Inami, Tokyo, Japan) within the 9-mm area marked with a 9-mm disposable biopsy punch (Kai medical, Tokyo, Japan). Then, the anterior chamber was irrigated with physiological saline.

### Intracameral injection of hiPSC-derived NCCs

Cell injection was performed using the corneal endothelial dysfunction model, which was created according to a previous method with a slight modification [26, 28]. Briefly, 200 μL aqueous humor was first extracted from the anterior chamber of the rabbit corneas. The hiPSC-derived NCCs or NIH-3T3 fibroblasts were suspended in 300 μL DMEM supplemented with 100 μM Y-27632 at a density of 1.0 × 10^6^ cells, and then, they were injected into the anterior chamber. After the injection, the animals were maintained with the operated eyes in the face-down position for 3 h under general anesthesia. For post-transplantation care, a 200-μL subconjunctival injection of a 1:1 mixture of 5 mg/mL dexamethasone sodium phosphate (Chenxin, Shandong, China) and 0.5 mg/mL atropine sulfate (Jinyao, Tianjin, China) was administered. Tobramycin dexamethasone eye ointment (Novartis, Basel, Switzerland) and an injection of the above-described mixture were administered once daily. Tobramycin and dexamethasone eye drops (Novartis) with 10 mM Y-27632 were also given topically three times a day [29]. This medication regime was maintained until the rabbits were sacrificed.

Corneal clarity and endothelial cell morphology were photographed using slit-lamp microscopy (Topcon, Tokyo, Japan) and the corneal density was evaluated by confocal microscopy (Heidelberg Engineering, Heidelberg, Germany). Corneal thickness was measured with a Handy Pachymeter (Tomey, Nagoya, Japan) and the mean of the measured values was then calculated (up to a maximum thickness of 1200 μm, which is the instrument’s maximum reading).

### Immunofluorescence staining

The differentiated cells were fixed with 4% paraformaldehyde (PFA) (Biosharp, Anhui, China) for 10 min, treated with 0.5%Triton X-100 (Beyotime Biotechnology, Shanghai, China) for 10 min, and blocked with 5% BSA (Boster Biological Technology, Wuhan, China) for 1 h at room temperature. Cornea whole-mount staining was performed as previously described [29]. The rabbits were sacrificed 7 days or 14 days after surgery and the operated eyes were enucleated. The whole cornea of each operated eye was fixed in 4% PFA for 12 min, incubated in 1% Triton X-100 for 10 min, and blocked with 2.5% BSA for 1 h at room temperature. Full-thickness corneal flat mounts were cut in half, and the samples including cells and rabbit corneas were treated with primary antibodies (Table 1) overnight at 4 °C and subsequently with Alexa Fluor 488- and 594-conjugated secondary antibodies (Invitrogen, Carlsbad, CA, USA) for 1 h at 37 °C. Staining for cytoskeleton F-actin was performed with phalloidin (Invitrogen) for 1 h at 37 °C. The nuclei were stained with 4,6-diamidino-2-phenylindole (DAPI) (Beyotime Biotechnology) and observed by fluorescence microscopy (Nikon, Japan, and Zeiss, LSM800, Germany).

**Table 1:** A list of antibodies used for immunostaining

Antibody	Supplier	Code	Dilution for staining
OCT4	Abcam	Ab18976	1:100
NANOG	Abcam	Ab80892	1:150
SOX2	Abcam	Ab137385	1:100
P75	Omnimabs	Om267105	1:150
TFAP2A	Santa Cruz	Sc-12726	1:150
TFAP2B	Abcam	Ab221094	1:100
SOX10	Abcam	Ab155279	1:100
PERIPHERIN	Absin	Abs130178	1:200
KERATOCAN	Bioss	Bs-11054R	1:100
COLLAGEN-I	Abcam	Ab34710	1:100
PHALLOIDIN	Life Tech	A12379	1:200
ZO-1	Invitrogen	40-2200	1:150
Na/K-ATPase	Abcam	Ab76020	1:200
SLC4A11	Bioss	Bs-13714R	1:200
α-SMA	Abcam	Ab32575	1:100
SNAIL2	CST	C19G7	1:200
Human Nuclei	Abcam	Ab191181	1:200
Donkey anti mouse, Alexa Fluor 488	Life Tech	A21202	1:200
Donkey anti rabbit, Alexa Fluor 488	Life Tech	A21206	1:200
Donkey anti mouse, Alexa Fluor 594	Life Tech	A21203	1:200

### Flow cytometry analysis

The differentiated cells were dissociated with 0.25% Trypsin-EDTA (Sigma). Briefly, 1 × 10^6^ cells were resuspended in phosphate-buffered saline (PBS) and incubated for 30 min at 4 °C with primary conjugated antibodies P75 (Biolegend, San Diego, CA, USA) and HNK-1 (Biolegend). Then, the cells were then subjected to flow cytometry using the BD FACS Calibur and analysis was performed using the Flow-Jo program (Treestar, OR, USA).

### Quantitative real-time reverse transcription polymerase chain reaction (qRT-PCR)

The hiPSC-derived NCCs and regenerated corneal endothelium were isolated for qRT-PCR analysis. Total RNA was extracted from the differentiated cells using the MiniBEST Universal RNA Extraction Kit (TaKaRa, Tokyo, Japan) at different time points after differentiation (days 0–7). The post-transplantation rabbit endothelium was retrieved by enucleating the eyeballs, washing them with PBS, and excising the corneas. Descemet’s membrane, together with the CECs, was stripped from each cornea using forceps, and the residual corneal stroma was washed with PBS to avoid CECs contamination. Total RNA was isolated from the post-transplanted rabbit endothelium using the TransZol Up Plus RNA Kit (TransGen Biotech, Beijing, China), according to the manufacturer’s protocol. A 400-ng sample of total RNA was reverse transcribed to complementary DNA with the Evo M-MLV RT Kit (TaKaRa), and qPCR analysis was performed using the SYBR Green qPCR Master Mix (Vazyme, Nanjing, China), according to the manufacturer’s protocol. The primers used in the qRT-PCR are listed in Table 2. Gene expression of the sample was normalized to glyceraldehyde-3-phosphate dehydrogenase (GAPDH).

**Table 2:** Primer sequences for quantitative reverse transcription transcription-polymerase chain reaction

Gene name	Forward	Reverse
*Human NANOG*	*TGGGCCTGAAGAAAACTATCCAT*	*GAAGTGGGTTGTTTGCCTTTG*
*Human OCT4*	*CCCGAAAGAGAAAGCGAACC*	*CCACACTCGGACCACATCC*
*Human SOX2*	*ACCCCTGGCATGGCTCTT*	*ATGCTGATCATGTCCCGGA*
*Human P75*	*CGACAACCTCATCCCTGTCTAT*	*CCACTGTCGCTGTGGAGTTTT*
*Human TFAP2B*	*CCGCCAAAGCCGTCTCT*	*TGGGTCGGCTGTTCCCT*
*Human TFAP2A*	*CCTGTCCAAGTCCAACAGCAAT*	*GACACTCGGGTGGTGAGAGC*
*Human SOX9*	*CCCCAACAGATCGCCTACAG*	*TCTGGTGGTCGGTGTAGTCGT*
*Human SOX10*	*CGCACCTGCACAACGCT*	*GCGGCCTTCCCGTTCTT*
*Human ZO-1*	*AGGATCCATATCCCGAGGAAA*	*CGAGGTCTCTGCTGGCTTGT*
*Human Na*^*+*^*/K*^*+*^*ATPase*	*CAGGGCAGTGTTTCAGGCTAA*	*TCGACGATTTTGGCGTATCTT*
*Human SLC4A11*	*GGACATCGCACGCAGGTT*	*CGTCATTGAGAGACCCGAAAG*
*Human AQP1*	*CCTCCAGCTGGTGCTATGC*	*AAGGACCGAGCAGGGTTAATC*
*Human N-cadherin*	*ATCCGACGAATGGATGAAAGA*	*ACTAACAGGGAGTCATATGGTGGA*
*Human COL8A2*	*CCGGCCACCTATACCTACGAT*	*TCCTGAAAAGGAGGAGTGGATGTA*
*Human P21*	*GACAGCAGAGGAAGACCATGTG*	*GGCGTTTGGAGTGGTAGAAATC*
*Human P16*	*TGCCCAACGCACCGAATA*	*GCACGGGTCGGGTGAGA*
*Human α-SMA*	*CAGAGACCCTGTTCCAGCCA*	*GCCCCCTGATAGGACATTGTTA*
*Human SNAIL2*	*GCGATGCCCAGTCTAGAAAATCT*	*CTTCTCCCCCGTGTGAGTTCTA*
*Human GAPDGH*	*CATGTTCGTCATGGGTGTGAA*	*CATGGACTGTGGTCATGAGTCCT*

### Senescence associated β-galactosidase staining

The senescence associated β-galactosidase staining (SA-β-Gal) staining was performed according to the manufacturer’s protocol (Cell Signaling Technology, Danvers, USA). The cornea was isolated 7 or 14 days after transplantation. The full-thickness corneal holder and the treated cells were fixed in 1× fixative (formaldehyde-glutaraldehyde mixture) for 15 min and then washed with PBS and incubated overnight in the β-Gal staining solution in a dry incubator at 37 °C. The cells were observed under a bright-field microscope (Leica BM5500B Microscope, Wetzlar, Germany).

### Statistical analysis

All the experiments were performed at least three times, and the data are presented as means ± standard deviation (SD). Statistical analysis was performed using SPSS 19.0 software (SPSS) and Prism 8 (GraphPad) software using the Student’s *t* test and ANOVA with Tukey’s HSD test. *p < 0.05, **p < 0.01, and ***p < 0.001 with specific comparisons are indicated in the figure legends.

## Results

### Inductive differentiation of NCCs from hiPSCs

**Fig. 1:**
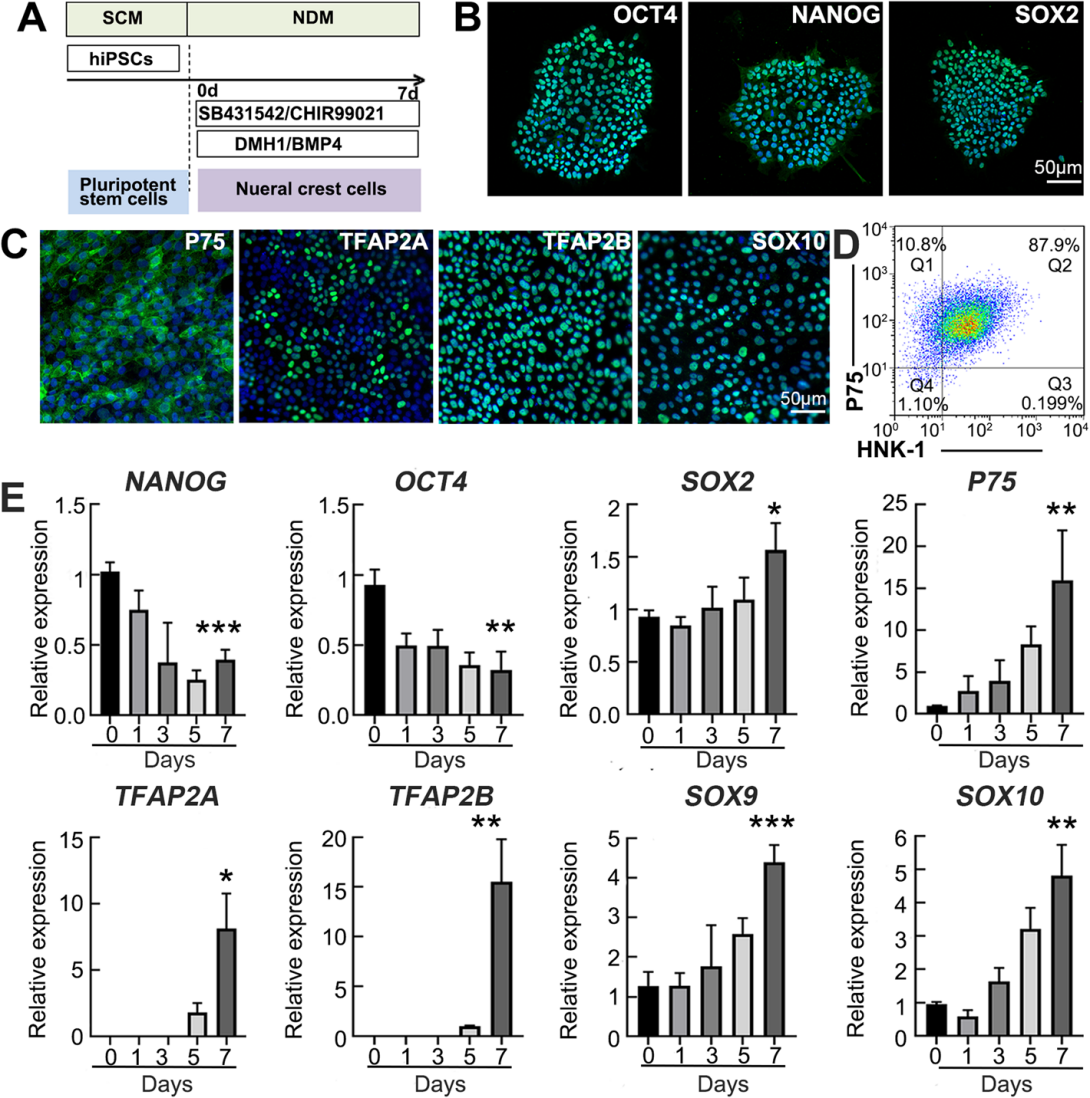
Inductive differentiation of hiPSCs into NCCs. **a** Schematic diagram of inductive differentiation. The hiPSCs were maintained in the SCM and induced the differentiation into NCCs in the NDM for 7 days. **b** Immunofluorescence staining of the hiPSCs before induction. **c** Immunofluorescence staining of the hiPSC-derived NCCs after 7 days of induction. **d** FACS analysis of P75 and HNK-1 dual-positive cells. **e** qRT-PCR analysis of the hiPSC-related genes (*NANOG*, *OCT4*, *SOX2*) and the NCC-related genes (*P75*, *TFAP2A*, *TFAP2B*, *SOX9*, *SOX10*). *P < 0.05, **P < 0.01, ***P < 0.001

For the induction of NCCs from hiPSCs, we modified the methods of GSK-3 and TGF-β inhibitors, as described previously [17, 23]. The hiPSCs were incubated in NDM containing 1 μM glycogen synthase kinase 3 (GSK-3) inhibitor CHIR99021, 2 μM transforming growth factor-beta (TGF-β) inhibitor SB431542, 15 ng/mL recombinant BMP4, and 1 μM BMP receptor inhibitor DMH1 for 7 days (Fig. 1a). The hiPSC U2 line was identified with positive staining of the pluripotent markers OCT4, NANOG, and SOX2 (Fig. 1b). After 7 days of induction, the hiPSC-derived NCCs were positive for the neural crest markers P75, TFAP2A, TFAP2B, and SOX10 (Fig. 1c), and the flow cytometry analysis confirmed that 81.8 ± 5.4% of the differentiated cells were dually positive for P75 and HNK-1 (Fig. 1d). More specifically, the expression of the pluripotent and neural crest-related genes was detected using qRT-PCR. As expected, expression of *NANOG* and *OCT4* showed an extremely decreasing trend, while the expression of *SOX2* was slightly elevated. The expression of the neural crest markers *P75*, *TFAP2A*, *TFAP2B*, *SOX9*, and *SOX10* displayed a continuous upregulated expression (Fig. 1e). These results suggest that hiPSCs can be differentiated into the homogenous neural crest cells using the modified protocol.

### Multi-lineage differentiation of hiPSC-derived NCCs in vitro

**Fig. 2:**
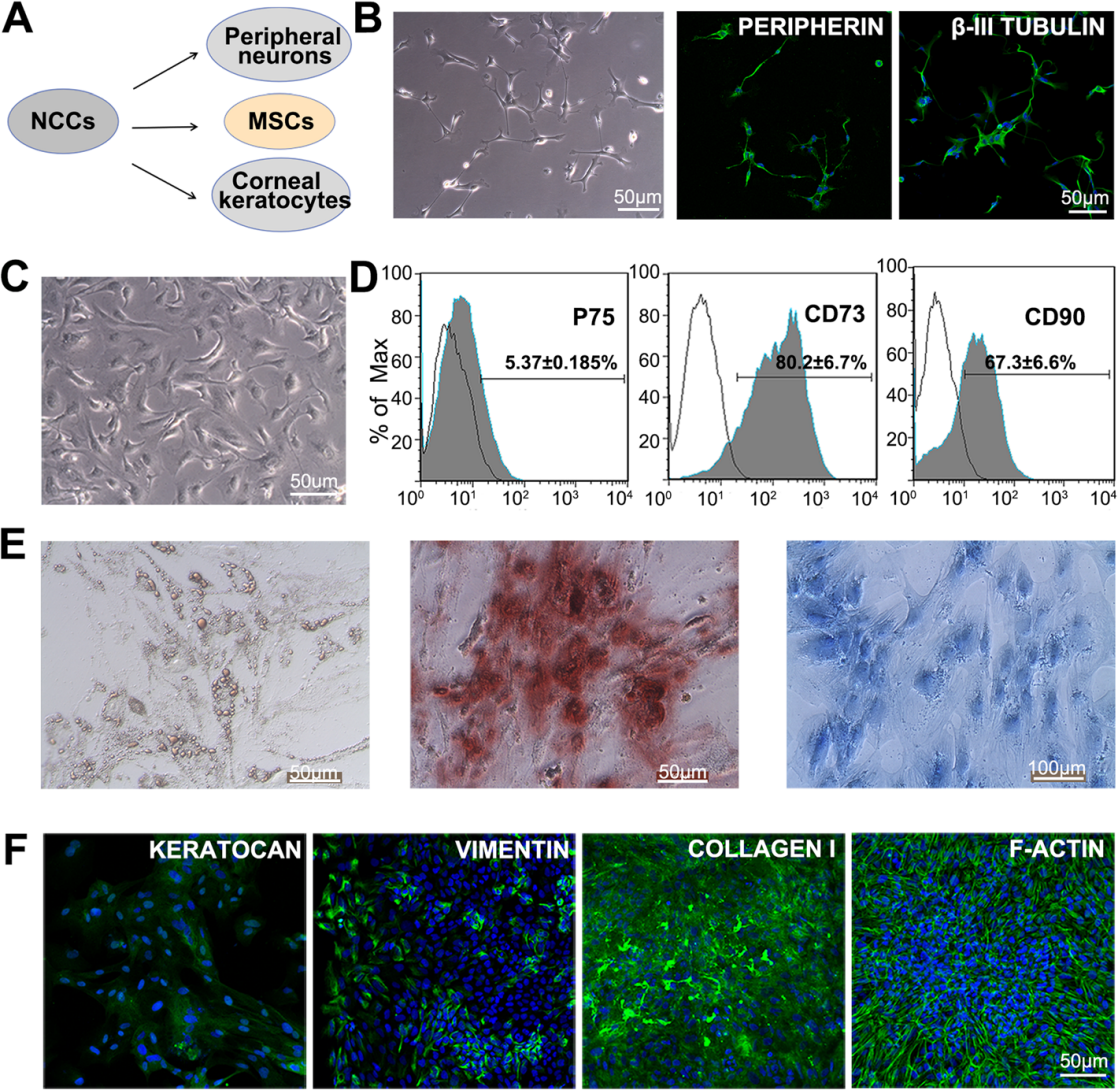
Multipotent characteristics of hiPSC-derived NCCs. **a** Schematic diagram of multipotent neural crest cells. **b** Morphology and immunofluorescence staining of the differentiated neurons. **c** Morphology of the differentiated MSCs. **d** FACS analysis of NCC-related marker P75 and MSC markers CD73 and CD90 in the differentiated MSCs. **e** Multipotent differentiation into adipocytes, osteocytes, and chondrocytes by Oil Red O staining, Alizarin Red S staining, and Alcian Blue staining, respectively. **f** Immunofluorescence staining of the differentiated corneal keratocytes

The neural crest is a multipotent and transient structure during vertebrate development, and it can give rise to several specialized subpopulations, including cranial, vagal, trunk, and cardiac NCCs [30]. We verified the multipotency of the hiPSC-derived NCC-like cells by inducing the NCCs to differentiate into peripheral neurons and mesenchymal derivatives, including adipocytes, chondrocytes, and osteocytes, as well as corneal keratocytes (Fig. 2a). When induced in the neuron differentiation medium for 10–14 days, the differentiated cells showed typical neurite outgrowth and positive staining for PERIPHERIN and β-III TUBULIN, indicating the acquisition of a peripheral neuron fate (Fig. 2b). When incubated in the MSC induction medium, the hiPSC-derived NCCs gradually changed into the spindle-shaped morphology (Fig. 2c). Flow cytometry analysis showed that the differentiated MSCs expressed CD73 and CD90 and lost the expression of P75 (Fig. 2d). Moreover, these cells could be successfully induced into adipocytes, osteocytes, and chondrocytes (Fig. 2e). When incubated in the keratocyte differentiation medium for 10 days, the hiPSC-derived NCCs acquired a typical morphology of corneal keratocytes and positive staining with the F-ACTIN, KERATOCAN, VIMENTIN, and COLLAGEN-I (Fig. 2f). Taken together, the results suggested that hiPSC-derived NCCs were capable of multi-lineage differentiation.

### Therapeutic outcome of the hiPSC-derived NCCs on rabbit corneal endothelial regeneration

**Fig. 3:**
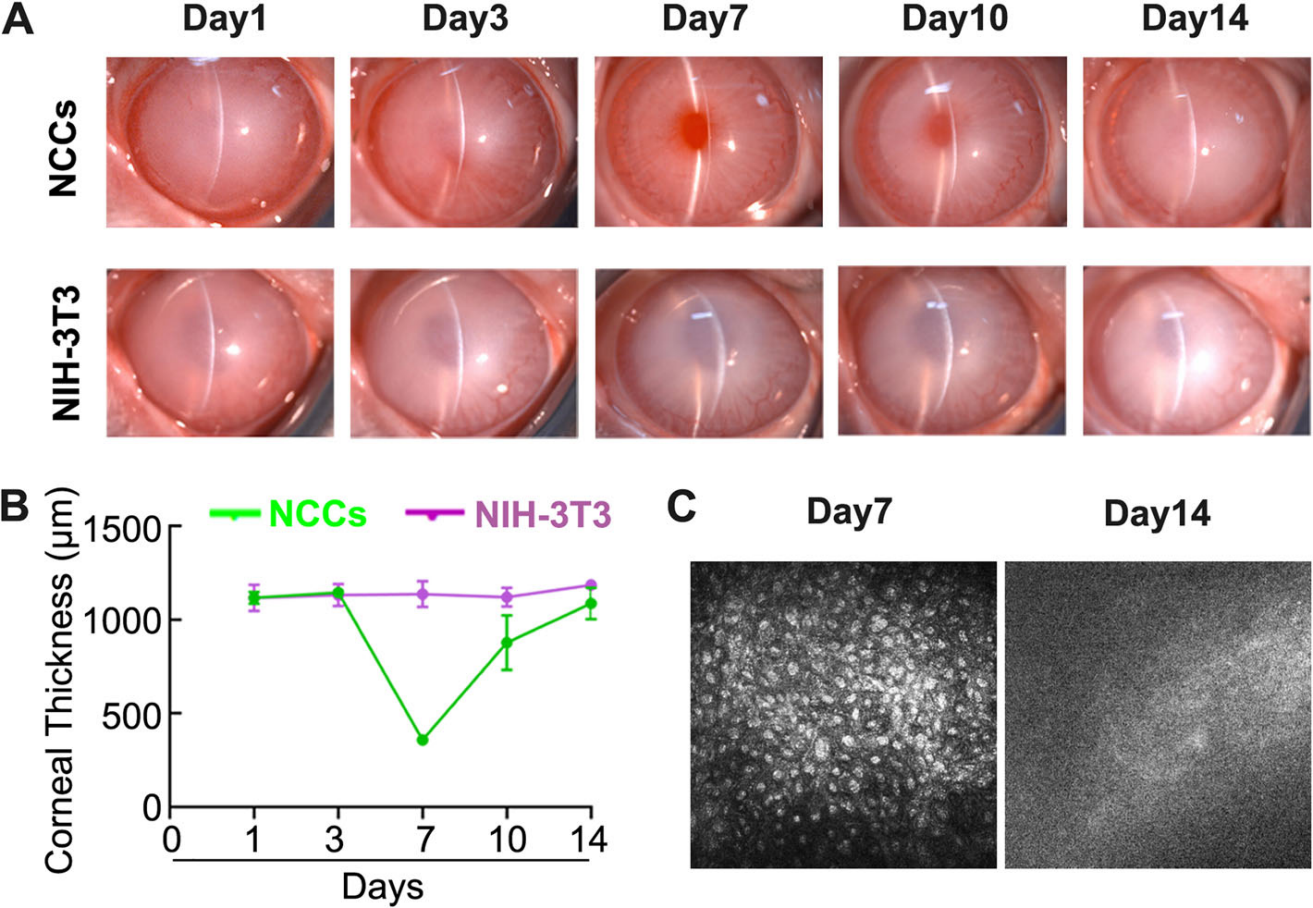
Intracameral injection of the hiPSC-derived NCCs in rabbits. **a** Slit-lamp photographs of rabbit corneas injected with either NIH-3 T3 fibroblasts or the hiPSC-derived NCCs. **b** Changes of corneal thickness after cell injection (*n* = 3 per group). **c** Representative images of corneal endothelium captured after 7 and 14 days of NCC group. Note the full coverage of polygonal cells at day 7 and blurred image at day 14

To evaluate their effect of corneal endothelial dysfunction, the differentiated cells (1.0 × 10^6^) with 100 μM Y-27632 were intracamerally injected into the anterior chamber of the rabbits following removal of corneal endothelium, with the NIH-3 T3 fibroblasts as control for injection. The control group showed severe opacity and edema throughout the follow-up to 14 days. However, the corneas of the rabbits injected with hiPSC-derived NCCs achieved a rapid improvement of corneal clarity within 7 days, but they exhibited the reappearance of corneal edema at day 10 and complete loss of clarity at day 14 (Fig. 3a). Consistently, the corneal thickness was gradually recovered to normal levels within 7 days, but it subsequently deteriorated to the similar levels as the rabbits with the injection of NIH-3 T3 fibroblasts at day 14 (Fig. 3b). Through the confocal microscopy, a regular arrangement of regenerated corneal endothelium appeared at 7 days of the hiPSC-derived NCC injection, but this had disappeared after 14 days due to corneal opacity (Fig. 3c). No other adverse effects, including abnormal accumulation of grafted cells and corneal neovascularization, were observed. Only slight inflammatory keratitis or anterior chamber exudation appeared after surgery, but rapidly disappeared with the use of anti-inflammatory eyedrops. These results suggest that the hiPSC-derived NCCs can rapidly recover corneal clarity and thickness after intracameral injection, but the long-term efficacy remains deteriorated and causes the recurrence of corneal edema.

### Characteristics of hiPSC-derived NCCs after intracameral injection

**Fig. 4:**
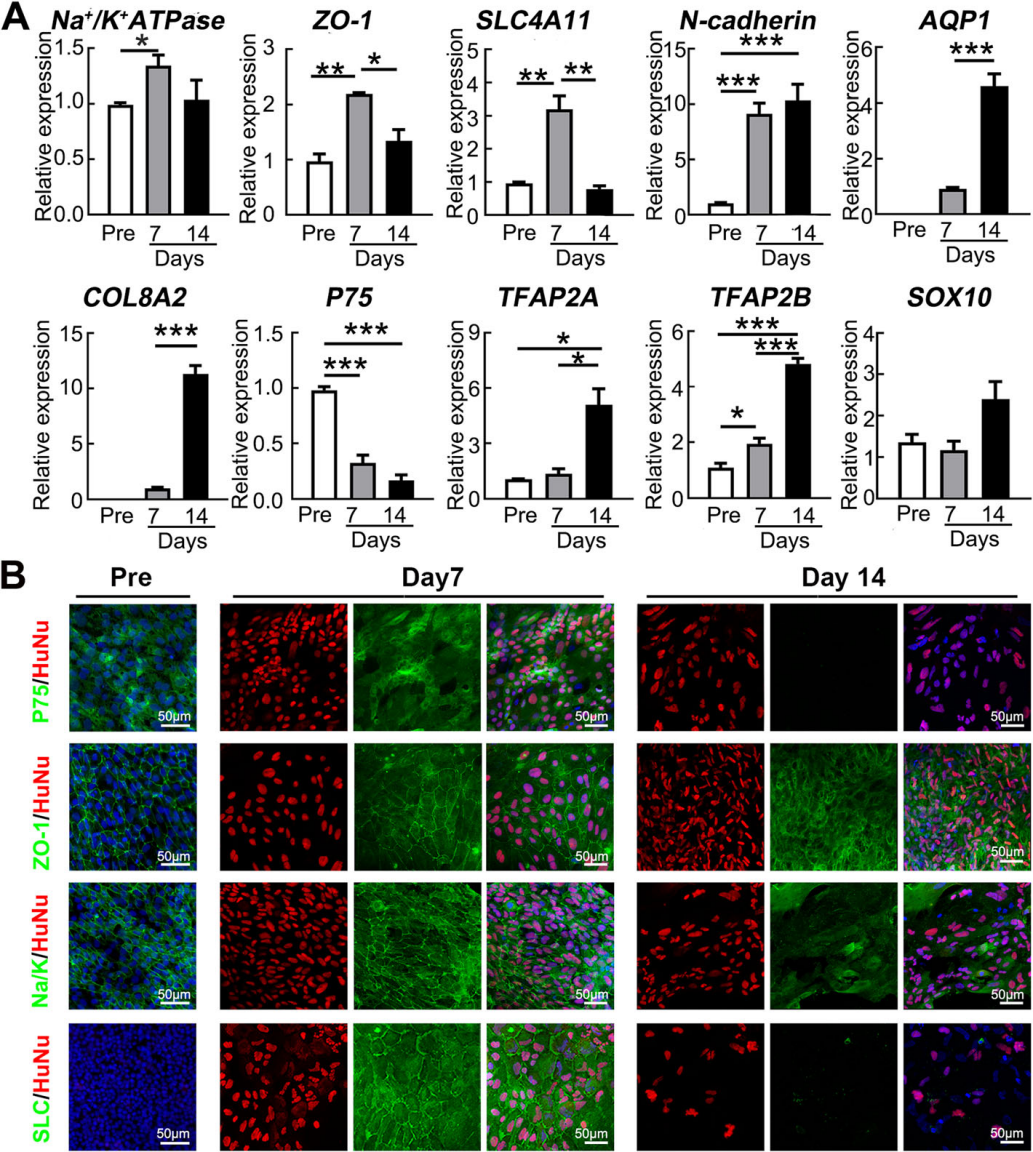
Improper maturation of the hiPSC-derived NCCs after intracameral injection. **a** qRT-PCR analysis of the corneal endothelial-related genes (*Na*^*+*^*/K*^*+*^*-ATPase*, *ZO-1*, *SLC4A11*, *AQP1*, *N-cadherin*, and *COL8A2*) and the NCC-related genes (*P75*, *TFAP2A*, *TFAP2B*, and *SOX10*) in the hiPSC-derived NCCs before transplantation (Pre), 7 days and 14 days after transplantation (*n* = 3), *P < 0.05, **P < 0.01, ***P < 0.001. **b** Immunofluorescence staining of the hiPSC-derived NCCs before transplantation, 7 days and 14 days after transplantation. DNA was visualized by staining with DAPI (blue) and Human Nuclei (HuNu)

To explore the characteristics changes of hiPSC-derived NCC functions in vivo*,* the transplanted NCCs were collected from the central corneal endothelium of the rabbits injected with NCCs for qRT-PCR and for whole-mount fluorescence staining at day 7 (clarity attainment) and day 14 (clarity loss). The qRT-PCR results showed that the expression levels of mature corneal endothelial markers and especially *ZO-1*, *SLC4A11*, and *N-cadherin* significantly increased at day 7 when compared with the pre-transplanted cells*.* In particular, the expressions of *AQP1* and *COL8A2* were hardly detected in the pre-transplanted cells but extremely upregulated in the post-transplanted cells at days 7 and 14. The expression of the neural crest-related gene *P75* was reduced significantly, while the expression of the corneal endothelial-specific transcription factors *TFAP2B* was increased and the expression of *SOX10* and *TFAP2A*, which was the verification of the cells derived from the neural crest, was continuous (Fig. 4a). Immunofluorescence staining was further performed to confirm the protein expression changes and cellular location. Before transplantation, the corneal endothelial genes Na+/K+-ATPase and ZO-1 clearly delineated the intercellular adhesion junctions and cellular boundaries of hiPSC-derived NCCs, but SLC4A11 was not expressed. After 7 days of transplantation, the injected cells assumed a hexagonal morphology and exhibited positive staining of ZO-1, Na^+^/K^+^-ATPase, and SLC4A11, with sporadic staining of the neural crest marker P75. However, after 14 days of transplantation, the injected cells became fibrotic morphology, accompanied with negative staining for P75 and SLC4A11, as well as the irregular arrangement of ZO-1 and Na^+^/K^+^-ATPase (Fig. 4b). These results suggest that the transplanted hiPSC-derived NCCs actually differentiate into CEC-like cells within the first 7 days, but they then undergo an improper maturation in the late stage of cell transplantation.

### EnMT and senescence of hiPSC-derived NCCs after intracameral injection

**Fig. 5:**
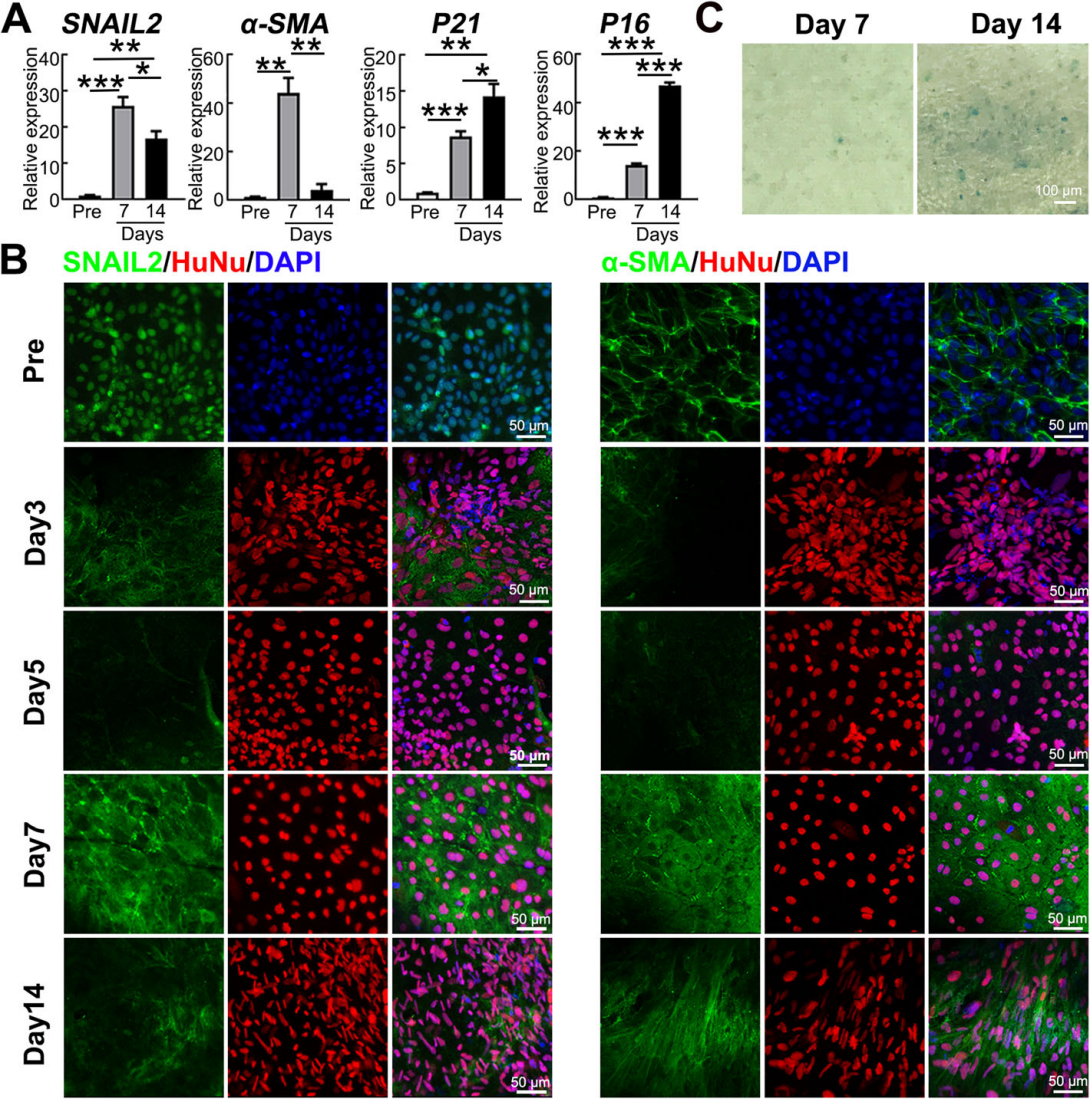
Fate-decision of hiPSC-derived NCCs after intracameral injection. **a** The qRT-PCR analysis results show the analysis of the EnMT-related genes (*α-SMA* and *SNAIL2*) and senescence-related genes (*P21* and *P16*) before transplantation (Pre), 7 days and 14 days after transplantation (*n* = 3), *P < 0.05, **P < 0.01, ***P < 0.001. **b** Immunofluorescence staining of the hiPSC-derived NCCs before transplantation, 7 days and 14 days after transplantation. DNA was visualized by staining with DAPI (blue) and Human Nuclei (HuNu). **c** SA-β-gal staining of the hiPSC derived-NCCs at day 7 and 14 after transplantation

The endothelial-mesenchymal transition and senescence have been reported as the typical changes of corneal endothelial dysfunction in human samples and animal models [31–33]. Therefore, we explored whether similar fate-decision appeared in the transplanted hiPSC-derived NCCs in rabbits. When compared with the pre-transplanted NCCs, the transplanted cells showed the significantly increased expression of the EnMT-related genes *SNAIL2* and *α-SMA* after 7 days. Although the EnMT-related mRNA transcripts decreased at day 14, the expression of *SNAIL2* was still higher than that of the pre-transplant cells (Fig. 5a). Immunofluorescence staining further confirmed the changing trend in SNAIL2 and α-SMA protein expression in the pre- and post-transplanted hiPSC-derived NCCs. The differentiated NCCs were positive for the EnMT markers SNAIL2 and α-SMA in vitro, although the RNA levels of these genes were low. After transplantation, most of the transplanted cells were negative for the expression of SNAIL2 and α-SMA on days 3 and 5. A number of positive cells increased at 7 days, but then decreased at 14 days, in agreement with the trend observed in the PCR results. Notably, SNAIL2 underwent a translocation from the nucleus to the cytoplasm after transplantation (Fig. 5b). The cell senescence markers *P16* and *P21* were highly upregulated in the transplanted NCCs on days 7 and 14. The central area of the regenerated corneal endothelium was positive for SA-β-Gal staining on days 7 and 14, with more obvious expression at day 14 (Fig. 5c). These results suggest that the recurrence of corneal edema in rabbits with NCC injection might be related to the EnMT and senescence of the transplanted NCCs in the later stage after transplantation.

## Discussion

Cultured cell transplantation represents an alternative pathway to relieve the severe limitation of human donor cornea. Previous reports have described the transplantation of cultured limbal epithelial cells [34], oral mucosal cells [35], hESC [36], and hiPSC-derived corneal epithelial cells for the treatment of limbal stem cell deficiency [37], as well as CECs for bullous keratopathy. In this study, we generated the hiPSC-derived neural crest cells and explored the outcome for corneal endothelial regeneration. Within 7 days after intracameral injection, the hiPSC-derived NCCs expressed typical endothelial markers and attained the normal corneal clarity and thickness. However, they subsequently underwent improper maturation, EnMT, and cellular senescence, which caused the recurrence of corneal edema within 14 days.

NCCs represent a transient cell population with stem cell-like properties that emerge from the dorsal neural plate border during vertebrate embryogenesis [38]. These cells migrate from the dorsal neural tube through epithelial to mesenchymal transition (EMT) after the closure of the neural tube and then migrate widely to contribute to a large variety of derivatives [38, 39]. Compared with terminally differentiated cells, hiPSC-derived NCCs tend to exhibit poor immunogenicity with low levels of HLA class I and no expression of HLA class II or positive costimulatory molecules against T cells [24]. In the present study, we induced hiPSCs into NCCs capable of multi-lineage differentiation into peripheral neurons, mesenchymal stem cells, and corneal keratocytes. Moreover, the hiPSC-derived NCCs did not form teratomas in NOD/SCID mice within 6 months of subcutaneous injection (data not shown). These findings suggest that the hiPSC-derived NCCs may provide an ideal source for the treatment of corneal endothelial dysfunction.

Previous studies have reported the in vitro induction of corneal endothelial-like cells derived from hESCs and hiPSCs [14, 40, 41], but the in vivo recovery outcome was unsatisfactory when transplantation in animal models of corneal endothelial dysfunction [41]. The most difficult is the lack of definitive specific markers to identify and enrich the differentiated CECs [42, 43]. Moreover, the differentiated cells may experience an incomplete maturation process through the in vitro induction, although they exhibited the positive staining of several corneal endothelial cell markers. The progenitor cells could be transplanted to repair the diseased tissues. Mechanistically, the therapeutic effects mostly depend on their in vivo maturation and functional integration [44, 45]. Consistently, here we confirmed that the hiPSC-derived NCCs actually differentiated into functional CECs in the first 7 days, which was accompanied by the decreased expression of neural crest-related genes and the increased expression of corneal endothelial-related gene. More importantly, the transplanted cells recovered normal corneal thickness and corneal clarity. These results suggest that the hiPSC-derived NCCs possess the capacity of differentiating into functional CECs within the microenvironment of anterior chamber. This is the first report of intracameral injection of hiPSC-derived NCCs into the rabbit model of corneal endothelial dysfunction, which avoids the process of the in vitro differentiation.

Unfortunately, the rabbits injected with NCCs showed the recurrence of cornea edema 10 days after the operation. According to the further comparison at 7 and 14 days after injection, we found that the transplanted cells underwent an improper maturation, EnMT, and cellular senescence. Most of the transplanted primary CECs experienced an abnormal maturation process due to the EnMT and ultimately exhibited a fibroblast-like morphology [26, 46]*.* Here, we observed a similar morphological alteration of the transplanted NCC-derived CEC-like cells from a polygonal morphology to a fibroblastic morphology, with a concomitant loss of the *Na*^*+*^*/K*^*+*^*-ATPase*, *ZO-1*, and *SLC4A11*. Differently, *COL8A2* expression significantly increased at day 14. The EnMT-like changes in the cultured human CECs could be attenuated by inhibiting the expression of COL8A2 [47]. The increase in *COL8A2* observed here might be associated with the EnMT process of the transplanted cells observed here at the later stage. Moreover, we found that the differentiated cells exhibited the peak expression of *SNAIL2* and *α-SMA* at day 7 at RNA level. When transplanted, SNAIL2 expression of the NCCs first decreased and appeared the translocation from nucleus to cytoplasm. The localization of SNAIL2 in the nucleus contributes to the stability of SNAIL2 and facilitates the induction of EMT, whereas SNAIL2 located in the cytoplasm reduces the stability and inhibits the EMT [48]. This just proved that the transplanted cells experienced the stage of directional differentiation and lost the characteristics of NCCs. In the later stage, the transplanted cells abnormally matured and the expression levels of SNAIL2 and SMA re-upregulated, which led to the occurrence of EnMT. Premature senescence of CECs has been implicated in Fuchs endothelial corneal dystrophy pathogenesis and the allograft after transplantation, which finally caused the loss of normal endothelial function [32, 49, 50]. Consistently, we found positive SA-β-Gal staining and increased expressions of P21 and P16 in the transplanted NCCs over time.

We suspect that this might be caused by the microenvironment of the host anterior chamber. The disease microenvironment was unfavorable for long-term survival, differentiation, functional engraftment, and integration of the transplanted cells [44, 51]. As well known, the aqueous humor contains multiple inflammatory mediators after corneal endothelial injury, such as IL-1β, TGF-β, and TNF-α, leading to the loss of normal endothelial function [52–54]. Consequently, the strategies to improve the host microenvironment, especially inhibiting the EnMT and cell senescence of the transplanted cells, are needed for the successful clinical application of hiPSC-derived NCCs.

## Conclusions

In summary, we successfully differentiated hiPSCs into NCCs using a simple and efficient method. We transplanted these hiPSC-derived NCCs into a rabbit model of corneal endothelial dysfunction and confirmed that the NCCs were capable of a rapid restoration of rabbit corneal thickness and clarity. However, the long-term recovery efficacy was impaired by the senescence and EnMT of the transplanted cells. Further studies are needed to achieve long-term therapeutic efficacy of hiPSC-derived NCCs for clinical applications.

## Data Availability

The data that support the findings of this study are available from the corresponding author upon request.

## Abbreviations

CECs: Corneal endothelial cells
hiPSCs: Human induced pluripotent stem cells
hESCs: Human embryonic stem cells
NCCs: Neural crest cells
hiPSC-derived NCCs: Human induced pluripotent stem cell-derived neural crest cells
EnMT: Endothelial-mesenchymal transition
SCM: Stem cell medium
DMEM: Dulbecco’s modified Eagle medium
FBS: Fetal bovine serum
PensStrep: Penicillin-streptomycin
NDM: Neural crest differentiation medium
DMEM/F12: Dulbecco’s modified Eagle medium/F12
BSA: Bovine serum albumin
FGF2: Fibroblast growth factor 2
BMP4: Bone morphogenetic protein 4
MSCs: Mesenchymal stem cells
ITS: Insulin-transferrin-selenium solution
MEM-NEAA: Non-essential amino acids solution
PFA: Paraformaldehyde
DAPI: 4,6-Diamidino-2-phenylindole
qRT-PCR: Quantitative real-time reverse transcription polymerase chain
GAPDH: Glyceraldehyde-3-phosphate dehydrogenase
SA-β-gal: Senescence-associated-β-galactosidase
SD: Standard deviation
GSK-3: Glycogen synthase kinase 3
TGF-β: Transforming growth factor-beta
EMT: Epithelial-mesenchymal transition
HuNu: Human nuclei
